# Anti-Vaccination

**Published:** 1895-09

**Authors:** Montague R. Leverson

**Affiliations:** Port Richmond (S. I.), N. Y.


### ANTI-VACCINATION.

PORT RICHMOND (S. I.), N. Y., June 18th, 1895.

TO THE PRESIDENT AND MEMBERS OF THE AMERICAN INSTITUTE OF HOMŒOPATHY:

DEAR SIR—I regret that the duties I have undertaken as Secretary of the Anti-Vaccination Society of America will prevent my attending the meeting of the Institute at Newport, but I ask you to kindly give some attention to the remarks I wish to lay before you.

I confess I am at a loss to understand how any person professing confidence in the teachings and principles of the great Hahnemann could ever give countenance to the blood-poisoning process of vaccination, nor yet how *any* surgeon who believes in *asepsis* can deliberately pour septic material into the blood. But the course of many persons calling themselves homœopaths, and even, I regret to say, some societies calling themselves homœopathic, has been so uncertain and erratic upon this subject that I feel impelled to beg your serious attention to it.

The testimony taken before the really *packed* tribunal of the Royal British Commission has nevertheless been so overwhelming in proof of the uselessness and injuriousness of vaccination that it is really impossible for any person of sound mind, who will take the trouble to study that testimony longer to entertain a doubt.

That commission was deliberately packed at its very outset, and it proceeded to take the evidence *in secret.* This was certainly wise, for had the press of England been permitted to lay before the people of that country the damaging evidence day by day as it proceeded, I verily believe the people would have risen in mobs and torn to pieces the blood-poisoners of the Local

Government Board—the analogue of our Boards of Disease, miscalled Boards of Health.

These things were established by that testimony without possibility of doubt, viz.:

1st—That vaccination never has prevented and never can prevent an attack of small-pox.

2d—That it is powerless to modify any such attack.

3d—That it has invaccinated, and is liable to invaccinate *syphilis, cancer, leprosy, tuberculosis, scrofula,* and many other diseases.

4th—That the human analogue of cow-pox is syphilis or great-pox. Proof of this last is also to be found in the classical works of Dr. Crookshank and Dr. Creighton, the latter the author of the article "Vaccination" in the *Encyclopedia Britannica,* 9th edition, volume XXIV.

5th—The testimony above referred to renders it almost certain that vaccination has caused more deaths and diseases than ever has small-pox, whose dangers and ravages have been wickedly exaggerated by official quacks.

6th—That Jenner was a mercenary charlatan whose ignorance and impatience of scientific methods were equalled only by his mendacity, in which last he has been imitated by his official followers.

In short, you will find, if you investigate the subject, that vaccination was conceived in ignorance, born in fraud and deception, and nourished and maintained by falsehood, robbery, and murder.

I earnestly entreat the American Institute of Homœopathy to appoint a committee to investigate this subject, and to report at its next annual meeting. I trust that the homœopathic body will do honor to itself by being one of the first official bodies to repudiate blood-poisoning as a medical process. I will cheerfully permit the members of your committee (should one be appointed), and any other member of the profession freely to consult at my office as above the four blue books of the British Royal Commission, the works of Drs. Crookshank and Creighton, also the classical works of Dr. Buckley on *Innocent Syphilis.* And, in conclusion, I desire you to bear in mind that every medical man who now opposes vaccination was brought up to believe in it, and that to adopt his present opinion he has had to overcome professional and educational prejudices of immense strength.

I have the honor to be, dear sirs,

Yours very truly,

MONTAGUE R. LEVERSON, M. D.

—*Antivaccination News.*

